# Effects of *Lactobacillus rhamnosus* DSM7133 on Intestinal Porcine Epithelial Cells

**DOI:** 10.3390/ani13193007

**Published:** 2023-09-24

**Authors:** Nikolett Palkovicsné Pézsa, Dóra Kovács, Fanni Somogyi, Zita Karancsi, Alma Virág Móritz, Ákos Jerzsele, Bence Rácz, Orsolya Farkas

**Affiliations:** 1 Department of Pharmacology and Toxicology, University of Veterinary Medicine Budapest, 1078 Budapest, Hungary; kovacs.dora@univet.hu (D.K.); somogyi.fanni4@gmail.com (F.S.); karancsi.zita@univet.hu (Z.K.); moritz.alma.virag@univet.hu (A.V.M.); jerzsele.akos@univet.hu (Á.J.); farkas.orsolya@univet.hu (O.F.); 2 National Laboratory of Infectious Animal Diseases, Antimicrobial Resistance, Veterinary Public Health and Food Chain Safety, University of Veterinary Medicine Budapest, 1078 Budapest, Hungary; 3 Department of Anatomy and Histology, University of Veterinary Medicine Budapest, 1078 Budapest, Hungary; racz.bence@univet.hu

**Keywords:** *Lactobacillus rhamnosus*, *Escherichia coli*, *Salmonella enterica* serovar Typhimurium, IPEC-J2, paracellular permeability, ROS, proinflammatory cytokines, adhesion

## Abstract

**Simple Summary:**

*Salmonella* species and *Escherichia coli* infections in pigs can result in substantial economic losses and frequently necessitate antibiotic treatment. The misuse of antibiotics has led to the emergence of antibiotic resistance, and residues in the human food chain may appear. It is pivotal for the swine industry to seek feed additives that can contribute to the health of the gastrointestinal tract. Probiotics are promising candidates for this purpose. Probiotic bacteria offer various advantageous effects, including the prevention of pathogen adhesion, the promotion of heat shock proteins, the modulation of cytokine production, antioxidative qualities, and the reinforcement of barrier function. The beneficial effect of probiotics is species/strain specific; the potential benefits need to be individually assessed for each probiotic strain or species. The objective of this study was to investigate the impact of *Lactobacillus rhamnosus* in in vitro porcine gastrointestinal infection models. Two economically significant swine pathogens, namely *Escherichia* and *Salmonella enterica* serovar Typhimurium, were applied to model gastrointestinal infections. *L. rhamnosus* demonstrated numerous advantageous effects, such as its antioxidative properties, ability to inhibit pathogens, anti-inflammatory characteristics, and capacity to enhance barrier function. This study makes a substantial contribution to the understanding of the specific advantageous effects associated with *L. rhamnosus* (which has relevance when choosing a probiotic in practice) and serves as a basis for further in vivo research.

**Abstract:**

Antimicrobial resistance is one of the biggest health challenges nowadays. Probiotics are promising candidates as feed additives contributing to the health of the gastrointestinal tract. The beneficial effect of probiotics is species/strain specific; the potential benefits need to be individually assessed for each probiotic strain or species. We established a co-culture model, in which gastrointestinal infection was modeled using *Escherichia coli* (*E. coli*) and *Salmonella enterica* serovar Typhimurium (*S. enterica* serovar Typhimurium). Using intestinal porcine epithelial cells (IPEC-J2), the effects of pre-, co-, and post-treatment with *Lactobacillus* (*L.*) *rhamnosus* on the barrier function, intracellular (IC) reactive oxygen species (ROS) production, proinflammatory cytokine (IL-6 and IL-8) response, and adhesion inhibition were tested. *E. coli*- and *S.* Typhimurium-induced barrier impairment and increased ROS production could be counteracted using *L. rhamnosus* (*p* < 0.01). *S.* Typhimurium-induced IL-6 production was reduced via pre-treatment (*p* < 0.05) and post-treatment (*p* < 0.01); increased IL-8 secretion was decreased via pre-, co-, and post-treatment (*p* < 0.01) with *L. rhamnosus*. *L. rhamnosus* demonstrated significant inhibition of adhesion for both *S.* Typhimurium (*p* < 0.001) and *E. coli* (*p* < 0.001 in both pre-treatment and post-treatment; *p* < 0.05 in co-treatment). This study makes a substantial contribution to the understanding of the specific benefits of *L. rhamnosus*. Our findings can serve as a basis for further in vivo studies carried out in pigs and humans.

## 1. Introduction

The human population is expected to reach 10 billion by 2060 [1]. This growing population correlates with an increasing need for food of animal origin, including pork meat [2]. In pigs, the efficient absorption of nutrients, better feed digestion, and, as a consequence of these factors, the desired growth performance requires a healthy gastrointestinal tract (GIT). Pathogens entering and colonizing the gastrointestinal tract might cause an imbalance in the microbial ecosystem, causing the inefficient absorption of nutrients and decreased growth performance, leading to economic losses for swine producers [3]. The weaning period is a crucial stage in the lives of piglets, as it involves a weakening of their immune system, rendering them more vulnerable to bacterial infections [4]. Post-weaning diarrhea is associated with enterotoxigenic *Escherichia coli* (ETEC) and is an economically significant disease worldwide [4]. Weaning pigs are also at risk of *Salmonella* spp. infection [5]. Clinical manifestations in ill pigs are enterocolitis, diarrhea, and dehydration; however, asymptomatic *Salmonella* infections are more common. Even if pigs successfully overcome the illness, they can still act as carriers and potentially release the bacteria for an extended period of several months [6]. Human health is further compromised if *E. coli* and *Salmonella* enter the food chain due to the zoonotic nature of these bacteria [7,8]. To treat gastrointestinal diseases, the swine industry relies on the use of antibiotics; however, their misuse in the past (often used for growth promoting purposes) led to residues appearing in the human food chain and the rise in antimicrobial resistance [3]. The latter issue is regarded as one of the most significant contemporary health challenges, and in accordance with the One Health concept, it is not only the veterinary field, but also humans and the shared natural environment, that are at risk [3,9]. Antibiotic usage for the promotion of growth was prohibited in the European Union in 2006 [10], and the new EU regulation (2019/6) further restricts the application of antibiotics in veterinary medicine [11]. On a global scale, pork consumption continues to rise, underscoring the importance of the swine industry’s quest to discover natural feed additives that can support the maintenance of a healthy gastrointestinal tract (GIT) and yield growth performance similar to what can be achieved using growth-promoting antibiotics. Probiotics are promising candidates for this purpose. Probiotics have undergone extensive examination through various in vitro [12,13,14,15] and in vivo [16,17,18] experiments, yet their precise mechanism of action still needs to be fully understood. Possible modes of action include (1) the competitive exclusion of pathogens to binding sites on epithelial cells, (2) prevention of pathogenic biofilm formation through the establishment of co-aggregates or auto-aggregates, (3) modulation of the host’s immune system (increased production of anti-inflammatory and decreased production of proinflammatory cytokines, elevated secretion of IgA, etc.), (4) enhancement of barrier function (induction of tight junctional protein expression, increased mucin production), (5) biodegradation or bioadsorption of toxins, and (6) production/induction of antimicrobial and/or antioxidant substances [3,19,20,21,22,23,24,25,26,27,28,29,30,31]. The majority of probiotic bacteria are part of the lactic acid-producing bacteria group and are derived from the intestine. Among the Lactobacillus genera, *L. brevis*, *L. casei*, *L. crispatus*, *L. farciminis*, *L. fermentum*, *L. murinus*, *L. gallinarium*, *L. paracasei*, *L. pentosus*, *L. plantarum*, *L. reuteri*, *L. rhamnosus*, and *L. salivarius* are frequently used in feed supplements [2,3]. *Lactobacillus rhamnosus* is one of the most studied probiotic bacteria. It has not been shown to be pathogenic and is sufficiently resistant to technological processes, which makes it an ideal probiotic [32]. Many positive effects of *L. rhamnosus* have been demonstrated in in vitro experiments. In murine (YAMC) and human (HT 29) intestinal epithelial cell cultures, *L. rhamnosus* strain GG (LGG) prevented cytokine (TNF, IL-1α and IFN-γ)-induced apoptosis of intestinal epithelial cells by activating the antiapoptotic protein kinase B enzyme and inhibiting the apoptotic p38 signal transduction pathway [33]. *Lactobacillus casei* subsp. *Rhamnosus* strain reduced the adhesion of enteropathogenic (EPEC) and enterotoxigenic (ETEC) *E. coli* and *Klebsiella pneumoniae* bacteria to the Caco-2 (human intestinal epithelial) cells, and its metabolites (present in the cell-free supernatant) showed bacteriostatic activity against nine bacterial species, including ETEC, EPEC, *S.* Typhimurium, *Shigella flexneri*, *Enterobacter cloacae*, *Pseudomonas aeruginosa*, *Enterococcus faecalis*, and *Clostridium difficile* (Forestier és mtsai, 2001) [32]. Two proteins produced by *L. rhamnosus* GG, namely p40 and p75, had a positive effect on tight junctional complexes in Caco-2 cells; hydrogen peroxide-induced tight junction (TJ) damage and, thus, cell permeability were reduced by modulating the protein kinase C and MAPK signaling pathways [34]. However, probiotics may also have adverse effects, such as immunocompromised states, D-lactic acidosis, and bacteremia. Some probiotics have natural resistance to certain antibiotics. This intrinsic antibiotic resistance is not transferable to other bacteria and might even be a useful tool when restoring the gut microbiota after antibiotic treatment. Repeated and long-term use of antibiotics may result in acquired resistance, which is a greater threat because it can be transferred from one bacterium to another, including pathogens and the intestinal microbiota [35].

In vitro cell culture systems allow the investigation of physiological and biochemical processes, offering reproducible and consistent experimental outcomes, aligning with the principles of the 3R concept, which advocates for the reduction in experiments conducted on animals [36]. The IPEC-J2 cell line is well-characterized and unique in two aspects: (1) it is non-transformed, and (2) it is derived from the small intestine. Single-cell monolayers are formed, exclusively consisting of epithelial cells [37]. Due to the parallels in size, weight, anatomy, and physiology between the pig and human intestine, the IPEC-J2 cell line serves as a valuable tool not only for mimicking the swine gastrointestinal tract, but also for drawing conclusions applicable to humans [38,39]. Its close resemblance to human physiology is particularly relevant when studying zoonotic enteric infections that can also impact humans. Furthermore, it facilitates the investigation of infections originating from pigs with a high degree of specificity [39,40]. The IPEC-J2 cell line is extensively employed for the examination of interactions with pathogens, such as *Salmonella enterica* and *E. coli*, as well as the assessment of the impacts of probiotic applications [12,14,41] and various other substances (for example, plant-derived substances) [42,43].

The primary objective of this study was to assess the potential positive impacts of the probiotic bacterium *L. rhamnosus* on the essential components of a healthy gastrointestinal tract using porcine intestinal epithelial (IPEC-J2) cells. We modeled gastrointestinal infections by utilizing two economically significant zoonotic swine pathogens, namely *E. coli* and *S. enterica* ser. Typhimurium (*S.* Typhimurium), which are responsible for a broad spectrum of gastrointestinal illnesses. Various treatment conditions (pre-treatment, co-treatment, and post-treatment) were incorporated into this study to assess the probiotics’ efficacy as preventive or therapeutic agents. *L. rhamnosus* enhanced barrier integrity, reduced inflammatory response, decreased intracellular (IC) reactive oxygen species (ROS) production, and inhibited the adhesion of pathogens.

## 2. Materials and Methods

### 2.1. IPEC-J2 Cell Line

Culture conditions of IPEC-J2 cells were described in detail by Schierack et al. In brief, cells were cultivated and sustained in a complete medium of Dulbecco’s Modified Eagle’s Medium and Ham’s F-12 Nutrient Mixture (DMEM/F12) and incubated at 37 °C in a humidified atmosphere with 5% CO_2_ [37]. For our experiments, cells with the passage numbers 49–52 were utilized. Cell viability was assessed by culturing cells on a 96-well plate; IL-6, IL-8, and intracellular ROS levels were determined with cells grown on 6-well culture plates. Adhesion inhibition assays were conducted with cells seeded on 24-well cell culture plates, and the paracellular permeability measurement was performed with cells cultured on 12-well polyester membrane cell culture inserts. All plates and inserts were obtained from Costar Corning Inc., Corning, NY, USA. In each instance, cells were cultured until they reached confluency. Cells were seeded onto the 96-well plates at a density of 0.01 × 10^6^ cells/well, 24-well plates at a density of 0.05 × 10^6^ cells/well, 12-well culture plates at a density of 0.1 × 10^6^ cells/well, and 6-well plates at a density of 0.3 × 10^6^ cells/well.

To eliminate any residual antibiotics before commencing the treatment of IPEC-J2 cells with bacterial suspensions (outlined in Section 2.2), the IPEC-J2 cells underwent two PBS washes; then, DMEM/F12 without antibiotics was added, and cells were incubated at 37 °C for 30 min.

### 2.2. Bacterial Culture

The bacterial strains used in our experiments were as follows: (1) The probiotic strain *Lactobacillus rhamnosus* DSM7133 was obtained from the Hungarian Dairy Experimental Institute Ltd. (2) *Salmonella* Typhimurium and (3) *E. coli* originated from gastrointestinal infections in pigs. The characterization of the applied *Salmonella* Typhimurium and *E. coli* strains and the methodology of preparing bacterial suspensions were previously described [44]. In brief, (I) microbeads were suspended in plain DMEM/F12 and (II) incubated for 18–24 h at 37 °C in 5% CO_2_/95% air atmosphere. In our experiments, we used *L. rhamnosus* suspensions in a concentration of 10^8^ CFU/mL and *E. coli* and *S.* Typhimurium suspensions in concentrations of 10^6^ CFU/mL.

### 2.3. Cell Viability Determination

The effect on cell viability of incubating IPEC-J2 cells with *L. rhamnosus* suspension for varying time periods was investigated using the neutral red uptake method, following the protocol described by Repetto et al. [45]. *L. rhamnosus* suspension of 10^8^ CFU/mL concentration was prepared as outlined in Section 2.2. IPEC-J2 cells were treated with *L. rhamnosus* suspension for 1 and 2 h at 37 °C with 5% CO_2_. As the control, treatment with plain medium for 1 h was used. After 24 h, the viability of the IPEC-J2 cells was assessed by measuring the absorbance at a wavelength of 540 nm using a Spectramax iD3 instrument (Molecular Devices, San Jose, CA, USA).

Our research team previously conducted experiments to assess the impact on the viability of IPEC-J2 cells when exposed to varying concentrations of *E. coli* and *S.* Typhimurium suspensions for different durations of incubation [43].

### 2.4. Research Design for Co-Culture Experiments

In our experimental procedures, IPEC-J2 cells underwent different treatments: **(1) pathogen exposure**: IPEC-J2 cells were exposed to either the *E. coli* or *S.* Typhimurium pathogen strains for 1 h, control cells were treated with plain DMEM/F12 medium, as positive controls, and IPEC-J2 cells exposed to either *E. coli* or *S.* Typhimurium were used; **(2) pre-treatment assay**: in pre-treatment experiments, cells were pre-incubated with *L. rhamnosus* for 1 h before adding the pathogen strain; **(3) co-treatment experiments**: co-treatment experiments involved the simultaneous addition of both the pathogen strain (*E. coli* or *S.* Typhimurium) and *L. rhamnosus* to IPEC-J2 cells; **(4) post-treatment assay**: following pathogen strain exposure (*E. coli* or *S.* Typhimurium), IPEC-J2 cells were incubated with *L. rhamnosus* for 1 h; **(5) mono-treatment assay**: IPEC-J2 cells were subjected to incubation with *L. rhamnosus*.

**Figure 1:**
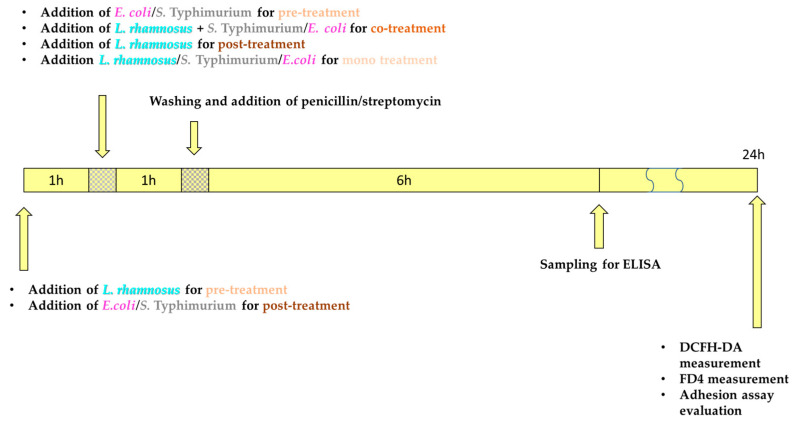
Chronological sequence used for experimental configuration. DCFH-DA: 2′,7′-dichloro-dihydro-fluorescein diacetate dye; FD4: fluorescein isothiocyanate–dextran tracer dye [46].

After these treatments, cells were rinsed with PBS, and DMEM/F12 supplemented with antibiotics, specifically 1% penicillin-streptomycin, was added to inhibit bacterial growth. Figure 1 illustrates the chronological sequence of our experimental arrangement.

### 2.5. Barrier Integrity Determination (FD4)

We evaluated the impact of *L. rhamnosus*, in combination with either *E. coli* or *S.* Typhimurium, on the paracellular permeability of IPEC-J2 cells. To assess this, we used fluorescein isothiocyanate–dextran (FD4) tracer dye obtained from Sigma-Aldrich, Darmstadt, Germany. Before the treatments, we measured the transepithelial electrical resistance (TEER) values of the IPEC-J2 cells to confirm the formation of a well-differentiated, fully confluent monolayer. Different treatments were performed as outlined in the research design section (Section 2.4). After the treatments, we rinsed the cells with PBS, and FD4 dissolved in phenol-free DMEM/F12 medium was added to the apical compartment at a final concentration of 0.25 mg/mL. To the basolateral chamber, we added phenol-free DMEM/F12 medium. The cells were then incubated at 37 °C with 5% CO_2_. After 24 h, from the basolateral compartment, 100 μL samples were collected. We measured the fluorescent signal using a Spectramax iD3 instrument (Molecular Devices, San Jose, CA, USA), with excitation at 485 nm and emission at 535 nm wavelengths. The experiment was performed three times using six replicates per treatment group.

### 2.6. IC ROS Level Determination (DCFH-DA)

We utilized the DCFH-DA method to assess how *L. rhamnosus* affected the intracellular production of ROS in IPEC-J2 cells [47]. Inflammation was induced in IPEC-J2 cells via exposure to *E. coli* (10^6^ CFU/mL) or *S.* Typhimurium (10^6^ CFU/mL). As outlined in the research design section (Section 2.4), the following treatments were conducted: mono-, pre-, co-, and post-treatments. Negative controls involved cells treated with plain medium, while positive controls consisted of cells treated with either *E. coli* or *S.* Typhimurium. Subsequent to the treatment, we removed the treatment solutions and replaced them with plain medium containing 1% penicillin–streptomycin.

For the detection procedure, the cells were first rinsed with PBS after 24 h. Subsequently, we added DCFH-DA reagent at a concentration of 40 mM to the cells. After one hour, we removed the reagent and performed two washes using 2 mL of phenol-free plain DMEM/F12. Following this step, the cells were scraped and lysed, and the resulting lysate was transferred into an Eppendorf tube and centrifuged at 4 °C and 4500 rpm for 10 min. We took 100 μL of supernatant from each sample and placed it into a 96-well plate. Using a Spectramax iD3 instrument from Molecular Devices, San Jose, CA, USA, we measured fluorescence at an excitation wavelength of 480 nm and an emission wavelength of 530 nm. This experiment was repeated three times, with each treatment group having six replicates.

### 2.7. Assessment of IL-6 and IL-8 Levels (ELISA)

Pre-, co-, and post-treatments were executed following the procedure outlined in the research design section (Section 2.4). After removing the treatment solutions, IPEC-J2 cells were incubated with cell culture medium, and cell supernatants were collected after 6 h. To determine IL-6 and IL-8 production, porcine-specific ELISA Kits (Sigma-Aldrich, Darmstadt, Germany) were employed, and the manufacturer’s instructions were followed. The sensitivity of the IL-6 ELISA kit was 45 pg/mL, and that of the IL-8 ELISA kit was 10 pg/mL. Intra-assay reproducibility was CV < 10% for both IL-6 and IL-8 kits, and inter-assay reproducibility was CV < 12% for both IL-6 and IL-8 kits. The experiment was conducted three times using six replicates per treatment group.

### 2.8. Bacterial Adhesion Assay

To assess the inhibitory effect of *L. rhamnosus* on *E. coli* or *S.* Typhimurium adhesion to IPEC-J2 cells, different treatments, as outlined in the research design section (Section 2.4), were applied. Cells solely treated with *S.* Typhimurium or *E. coli* were used as controls. IPEC-J2 cells were incubated for 1 h with the bacterial suspensions and then washed to remove any unbound bacteria. Subsequently, the cells were lysed using 500 µL of 0.1% Triton X-100. Viable counts of *S.* Typhimurium and *E. coli* were assessed via serial dilution and plating on ChromoBio Salmonella Plus Base (for *S.* Typhimurium) or ChromoBio Coliform (for *E. coli*) agar. These selective agars were purchased from Biolab Zrt. (Budapest, Hungary). We calculated adhesion as a percentage relative to the control. The adherence of both *E. coli* and *S.* Typhimurium was normalized against the control [44]. The experiment was conducted three times, with each treatment group having four replicates.

### 2.9. Statistical Analysis

To enable data comparability with other measurements, a control percentage (%) was calculated. The mean concentration of the control cells served as the reference point at 100%, against which values from various treatment groups were compared. For all measured parameters, mean values and standard deviations (SD) were computed across all treatment groups. We assessed data normality and variance homogeneity through diagnostic tests using the R 4.0.4 software package developed by R Foundation for Statistical Computing, Vienna, Austria. In each instance, both criteria were met, allowing us to analyze differences between the mean values of distinct experimental groups using one-way ANOVA and Tukey’s post hoc test using R 4.0.4 software. The significance was interpreted if the *p*-value fell below 0.05. To evaluate cell viability, IC ROS, IL-6/IL-8, and barrier integrity, six replicates per treatment group were utilized, while for adhesion inhibition determination, four replicates per treatment group were applied [46].

## 3. Results

### 3.1. Cell Viability Determination

**Figure 2:**
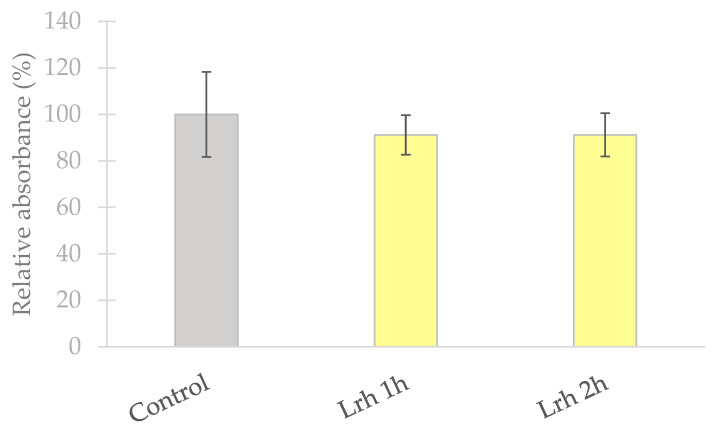
**IPEC-J2 cell’s viability following exposure to *Lactobacillus rhamnosus* (Lrh) for durations of 1 and 2 h. Control**: IPEC-J2 cells were treated with plain cell culture medium for 1 h. **Lrh 1 h**: IPEC-J2 cells were exposed to *L. rhamnosus* (Lrh) suspension at a concentration of 10^8^ CFU/mL for 1 h. **Lrh 2 h**: IPEC-J2 cells were exposed to *L. rhamnosus* (Lrh) suspension at a concentration of 10^8^ CFU/mL for 2 h. The data are shown as mean values, along with their corresponding standard deviations, and represented as relative absorbance, with the mean of the control group set at 100%. *n* = 6/group [46].

The viability of IPEC-J2 cells remained unaffected when subjected to a 10^8^ CFU/mL concentration of *L. rhamnosus* suspension for 1 and 2 h, showing no significant alterations compared to the control group (Figure 2) [46].

### 3.2. Barrier Integrity Assessment

**Figure 3:**
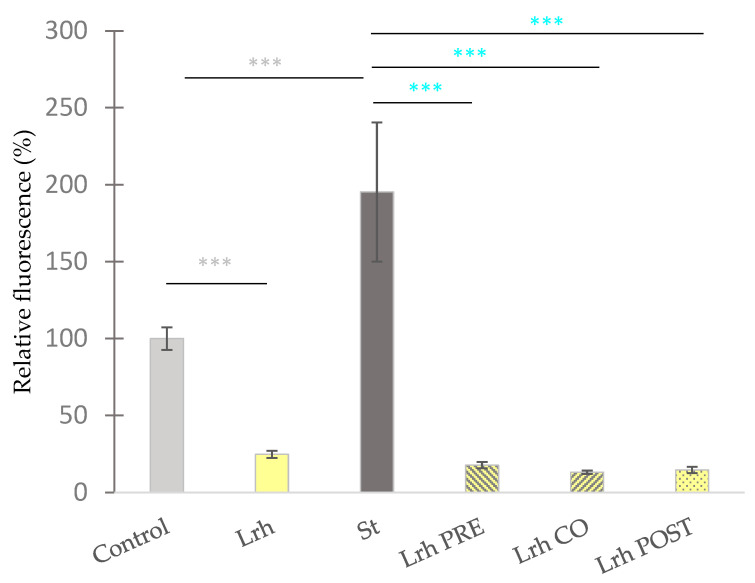
**Paracellular permeability of IPEC-J2 cells after treatment with *S.* Typhimurium and *L. rhamnosus* (Lrh). Control**: treatment with plain medium; **Lrh**: treatment with 10^8^ CFU/mL of *L. rhamnosus*; **St**: treatment with 10^6^ CFU/mL of *S.* Typhimurium; **Lrh PRE**: pre-treatment before *S.* Typhimurium infection with 10^8^ CFU/mL of *L. rhamnosus*; **Lrh CO**: co-treatment of *S.* Typhimurium infection with 10^8^ CFU/mL of *L. rhamnosus*; **Lrh POST**: treatment after *S.* Typhimurium infection with 10^8^ CFU/mL of *L. rhamnosus*. The data are presented as mean values, along with their corresponding standard deviations, and represented as relative fluorescence, with the mean of the control group set at 100%. *n* = 6/group. Significant difference: *** *p* < 0.001, in grey, compared to the control. *** *p* < 0.001, in turquoise, compared to *S.* Typhimurium treatment [46].

**Figure 4:**
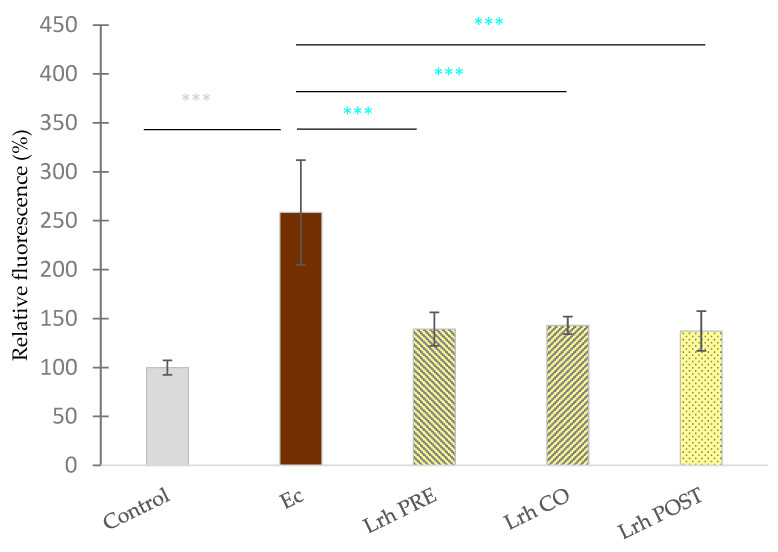
**Paracellular permeability of IPEC-J2 cells after treatment with *E.coli* and *L. rhamnosus* (Lrh). Control**: treatment with plain medium; **Ec**: treatment with 10^6^ CFU/mL of *E. coli*; **Lrh PRE**: pre-treatment before *E. coli* infection with 10^8^ CFU/mL of *L. rhamnosus*; **Lrh CO**: co-treatment of *E. coli* infection with *L. rhamnosus* 10^8^ CFU/mL; **Lrh POST**: treatment after *E. coli* infection with 10^8^ CFU/mL of *L. rhamnosus*. The data are presented as mean values, along with their corresponding standard deviations, and represented as relative fluorescence, with the mean of the control group set at 100%. *n* = 6/group. Significant difference: *** *p* < 0.001, in grey, compared to the control. *** *p* < 0.001, in turquoise, compared to *E. coli* treatment [46].

Following a 24-h period of exposure to the pathogen, the epithelial cell layer showed partial disruption. A significant increase in fluorescence intensity in the basolateral compartment was measured—compared to the untreated control samples—when IPEC-J2 cells were treated with *S.* Typhimurium (*p* < 0.001) (Figure 3) or *E. coli* (*p* < 0.001) (Figure 4).

Compared to the control group, the sole treatment with *L. rhamnosus* resulted in a significant decrease (*p* < 0.001) in the fluorescence intensity (Figure 3). Pre-, co-, and post-treatment with *L. rhamnosus* led to a significant decrease in the FD4 tracer in the basolateral chamber if cells were exposed to *S.* Typhimurium (Figure 3) or *E. coli* (Figure 4) (*p* < 0.001) [46].

### 3.3. Intracellular Reactive Oxigen Species Level Assessment

**Figure 5:**
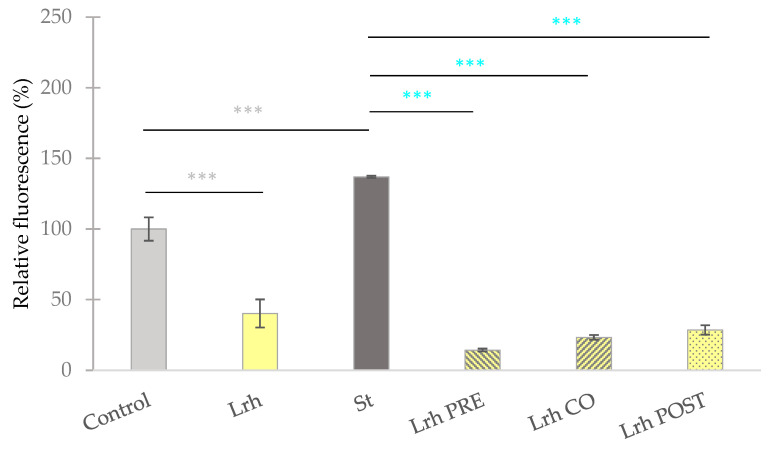
**The level intracellular reactive oxygen species in IPEC-J2 cells after treatment with *S.* Typhimurium (St) and *L. rhamnosus* (Lrh). Control**: treatment with plain medium; **Lrh**: treatment with 10^8^ CFU/mL of *L. rhamnosus*; **St**: treatment with 10^6^ CFU/mL of *S.* Typhimurium; **Lrh PRE**: pre-treatment before *S.* Typhimurium infection with 10^8^ CFU/mL of *L. rhamnosus*; **Lrh CO**: co-treatment of *S.* Typhimurium infection with 10^8^ CFU/mL of *L. rhamnosus*; **Lrh POST**: treatment after *S.* Typhimurium infection with 10^8^ CFU/mL of *L. rhamnosus*. The data are presented as mean values, along with their corresponding standard deviations, and represented as relative fluorescence, with the mean of the control group set at 100%. *n* = 6/group. Significant difference: *** *p* < 0.001, in grey, compared to the control. *** *p* < 0.001, in turquoise, compared to *S.* Typhimurium treatment [46].

**Figure 6:**
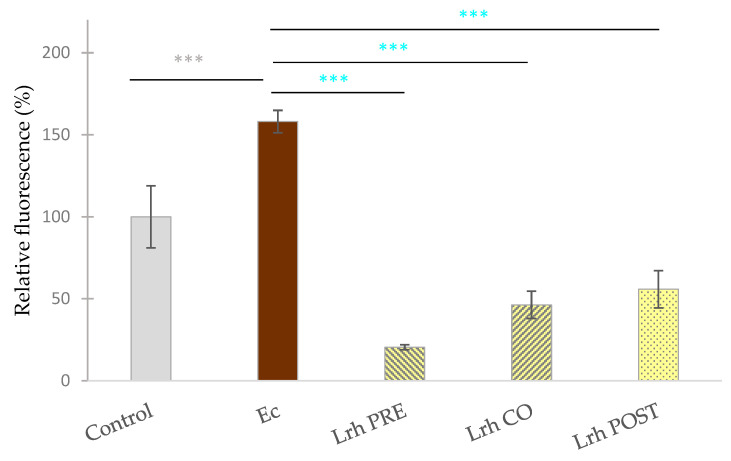
**The level intracellular reactive oxygen species in IPEC-J2 cells after treatment with *E. coli* (Ec) and *L. rhamnosus* (Lrh). Control**: treatment with plain medium; **Ec**: treatment with *E. coli* 10^6^ CFU/mL; **Lrh PRE**: pre-treatment before *E. coli* infection with 10^8^ CFU/mL of *L. rhamnosus*; **Lrh CO**: co-treatment of *E. coli* infection with 10^8^ CFU/mL of *L. rhamnosus*; **Lrh POST**: treatment after *E. coli* infection with 10^8^ CFU/mL of *L. rhamnosus*. The data are presented as mean values, along with their corresponding standard deviations, and represented as relative fluorescence, with the mean of the control group set at 100%. *n* = 6/group. Significant difference: *** *p* < 0.001, in grey, compared to the control. *** *p* < 0.001, in turquoise, compared to *E. coli* treatment [46].

Exposure to *S.* Typhimurium (Figure 5) or *E. coli* (Figure 6) led to a significant increase in the fluorescence compared to the control (*p* < 0.001). Conversely, when IPEC-J2 cells were solely treated with *L. rhamnosus*, a reduction in fluorescence was observed in comparison to the control (*p* < 0.001) (Figure 5). Pre-treatment, co-treatment, and post-treatment with *L. rhamnosus* and *S.* Typhimurium resulted in a marked decrease in ROS levels compared to cells exclusively challenged by *S.* Typhimurium (*p* < 0.001), as depicted in Figure 5. Similar observations were made when IPEC-J2 cells were exposed to *E. coli*, where all three treatment combinations resulted in diminished ROS levels, in contrast to samples solely treated with *E. coli* (*p* < 0.001) (Figure 6) [46].

### 3.4. Interleukin Level Assessment

**Figure 7:**
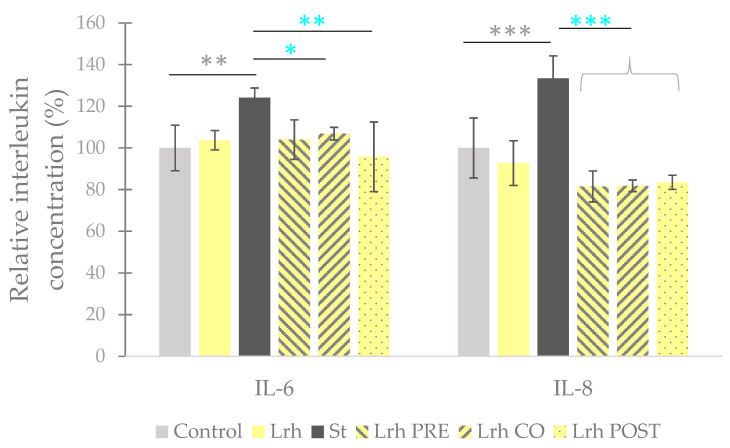
**Interleukin (IL-6 and IL-8) levels of IPEC-J2 cells after treatment with *S.* Typhimurium (St) and *L. rhamnosus* (Lrh). Control**: treatment with plain medium; **St**: treatment with 10^6^ CFU/mL of *S.* Typhimurium; **Lrh**: 10^8^ CFU/mL of *L. rhamnosus*; **Lrh PRE**: pre-treatment before *S.* Typhimurium infection with 10^8^ CFU/mL of *L. rhamnosus*; **Lrh CO**: co-treatment of *S.* Typhimurium infection with 10^8^ CFU/mL of *L. rhamnosus*; **Lrh POST**: treatment after *S.* Typhimurium infection with 10^8^ CFU/mL of *L. rhamnosus*. The data are presented as mean values, along with their corresponding standard deviations, and represented as relative interleukin concentration, with the mean of the control group set at 100% (122.6 pg/mL for IL-6 and 36.33 pg/mL for IL-8). *n* = 6/group. Significant difference: ** *p* < 0.01, *** *p* < 0.001 in grey, compared to the control. * *p* < 0.05; ** *p* < 0.01; *** *p* < 0.001, in turquoise, compared to *S.* Typhimurium treatment [46].

**Figure 8:**
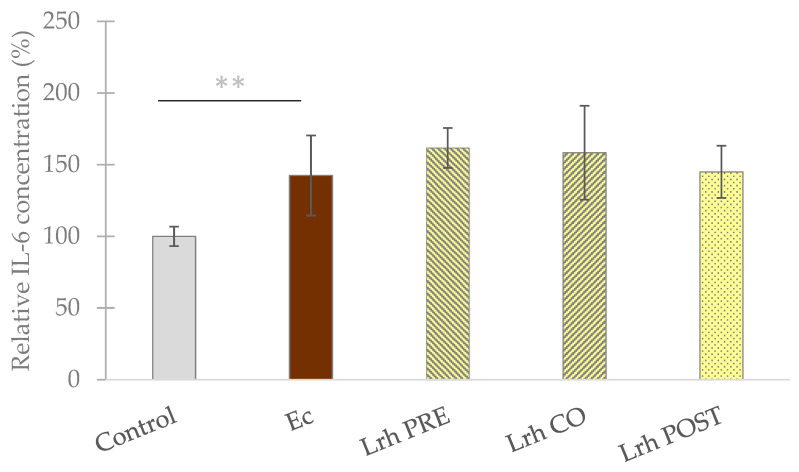
**Interleukin-6 levels of IPEC-J2 cells after treatment with *E. coli* (Ec) and *L. rhamnosus* (Lrh). Control**: treatment with plain medium; **Ec**: treatment with 10^6^ CFU/mL of *E. coli*; **Lrh PRE**: pre-treatment before *E. coli* infection with 10^8^ CFU/mL of *L. rhamnosus*; **Lrh CO**: co-treatment of *E. coli* infection with 10^8^ CFU/mL of *L. rhamnosus*; **Lrh POST**: treatment after *E. coli* infection with 10^8^ CFU/mL of *L. rhamnosus*. The data are presented as mean values, along with their corresponding standard deviations, and represented as relative IL-6 concentration, with the mean of the control group set at 100% (130.2 pg/mL). *n* = 6/group. Significant difference: ** *p* < 0.01, in grey, compared to the untreated control [46].

Challenging IPEC-J2 cells with *S.* Typhimurium resulted in a significant elevation in the secretion of IL-6 (*p* < 0.05) and IL-8 (*p* < 0.001) (Figure 7) compared to uninfected control cells. *E. coli* infection also induced a significant increase in IL-6 secretion compared to the control cells (*p* < 0.05) (Figure 8). Conversely, when IPEC-J2 cells were solely treated with *L. rhamnosus*, no significant change in the secretion of IL-6 or IL-8 (compared to the control) was elicited (Figure 7). Both pre-treatment and post-treatment with *L. rhamnosus* led to a significant reduction (*p* < 0.05 for Lrh PRE and *p* < 0.01 for Lrh POST) in IL-6 production compared to the levels induced by *S.* Typhimurium. However, co-treatment with *L. rhamnosus* did not result in a decrease in IL-6 production compared to the levels induced by *S.* Typhimurium (Figure 7). Pre-, co-, and post-treatment with *L. rhamnosus* significantly reduced the IL-8 production of IPEC-J2 cells compared to the levels induced by *S.* Typhimurium (*p* < 0.001) (Figure 7). Conversely, none of the treatment combinations could cause any significant alteration in the IL-6 secretion evoked by *E. coli* (Figure 8) [46].

### 3.5. Bacterial Adhesion Assessment

Treatment combinations with *L. rhamnosus* effectively inhibited the adhesion of both *E. coli* (*p* < 0.001 for pre-treatment and post-treatment; *p* < 0.05 for co-treatment) and *S.* Typhimurium (*p* < 0.001). When IPEC-J2 cells were exposed to *S.* Typhimurium, adding *L. rhamnosus* prior to or at the same time as the pathogen infection resulted in nearly identical inhibitory effects on *S.* Typhimurium adhesion, while post-treatment showed slightly reduced effectiveness. Specifically, *S.* Typhimurium adhesion was reduced by 96.33% via pre-treatment, 96.76% via co-treatment, and 91.02% via post-treatment. In the case of *E. coli* exposure, pre-treatment exhibited the most pronounced inhibitory effect, while co-treatment was the least effective. Precisely, *E. coli* adhesion was reduced by 90.80% via pre-treatment, 34.92% via co-treatment, and 74.63% via post-treatment (Table 1) [46].

## 4. Discussion

Discovering alternative feed additives that can support gastrointestinal tract health without resorting to antibiotics has emerged as a crucial concern across all sectors involved in food animal production, including the swine industry [48]. Research has demonstrated that probiotics can have positive impacts on the prerequisites of a well-functioning GIT; however, their effect is strain/species dependent [13,26,28,49,50]. Little emphasis is given to the species- and disease-specific effects of probiotics; however, this issue could explain why not all probiotics are equally effective in some types of diseases [51]. Hence, it is essential to evaluate the potential benefits of each individual probiotic strain or species. In this research, we examined the particular advantageous effects of porcine-derived *L. rhamnosus* as a possible feed supplement that could enhance the gastrointestinal tract, potentially preventing or aiding the treatment of bacterial infections in the intestines of swine. Our study was the first study to thoroughly investigate the particular impact of *L. rhamnosus* on intracellular ROS production, the secretion of cytokines IL-6 and IL-8, and paracellular permeability, as well as its ability to inhibit the adhesion of *E. coli* and *S.* Typhimurium using an in vitro epithelial cell model of porcine origin. We hypothesized that *L. rhamnosus* might (1) strengthen epithelial integrity, (2) decrease IL-6 and IL-8 secretion, (3) reduce the amount of IC ROS, and (4) inhibit the adhesion of *S.* Typhimurium and *E. coli*. IPEC-J2 cells were exposed to two swine pathogens of significant economic importance known to trigger various gastrointestinal diseases in pigs: *S.* Typhimurium and *E. coli* [8,49,52,53].

Intestinal barrier function can be enhanced using probiotics; the reduction in TJ proteins induced by LPS (lipopolysaccharide) could be reversed through pre-treatment with *L. reuteri* I5007 or its culture supernatant in IPEC-J2 cells. Moreover, *L. reuteri* I5007 elevated the presence of TJ proteins (claudin-1, occludin, and zonula occludens-1) in neonatal piglets [50]. In our experiments, the pathophysiological challenge induced by *E. coli* or *S.* Typhimurium led to a notable rise in the quantity of FD4 dye detected in the basolateral compartment. This observation suggests that these strains had the capacity to compromise the barrier’s integrity, consistent with earlier research findings [54]. LPS or bacterial metabolites, including secreted effector molecules and bacterial surface proteins, could potentially be accountable for the impairment of the epithelial barrier. Pathogens could also trigger enterocyte apoptosis or lead to the opening of the paracellular permeation pathway, possibly due to alterations in or the displacement of TJ or cytoskeletal proteins. This issue could lead to elevated TEER values, indicating the impairment of barrier integrity [55]. In our experiments, we observed that treatment with only *L. rhamnosus* led to a reduction in paracellular permeability. This finding aligns with previous research indicating that the use of probiotics by themselves may either have no impact on the integrity of the epithelial barrier or potentially enhance its function [55,56,57,58,59,60]. *Enterococcus faecium* per se enhanced the barrier function of Caco-2 cells [55]. Our experiments revealed that when *L. rhamnosus* was added before, at the same time, or after the exposure to *E. coli* or *S.* Typhimurium, it effectively prevented the detrimental effects on barrier integrity and significantly decreased the FD4 flux. Research conducted into Caco-2 and T84 cells has also demonstrated that probiotic strains, such as *L. Plantarum*, *L. Acidophilus*, or *L. Rhamnosus*, could counteract the barrier-impairing effects of *E. coli* [60,61].

To verify the antioxidant impact of utilizing *L. rhamnosus* before, during, and after treatment, we assessed the ability of the treatment methods to mitigate the generation of reactive oxygen species. In our experiments, *S.* Typhimurium and *E. coli* triggered an IC ROS burst in IPEC-J2 cells, which could be significantly diminished through the prior, simultaneous, or subsequent addition of *L. rhamnosus*. Thus, *L. rhamnosus* has powerful antioxidant properties against pathogen challenge. Using the DCFH-DA method allows us to assess total ROS production. Consequently, our findings indicate a universal reduction in ROS levels due to *L. rhamnosus*. Furthermore, this reduction was not influenced by the specific source of oxidative stress, whether it was *E. coli* or *S.* Typhimurium. Nonetheless, it remains unclear whether the reduction in ROS can be attributed to the probiotic bacteria itself or compounds generated by the probiotics. Our result aligns with previous research, in which the antioxidant attributes of probiotics were demonstrated. Specifically, in IPEC-J2 cells, the favorable impact of *L. plantarum* ZLP001 on ROS production has been verified, while in the IPEC-1 cell line, *Bacillus amyloliquefaciens* SC06 was effective in mitigating H_2_O_2_-induced oxidative stress [62]. To understand the exact antioxidant mechanism of action of *L. rhamnosus*, further research would be needed to investigate the effects of *L. rhamnosus* on different ROS (e.g., superoxide anion radical, hydrogen peroxide, and hydroxyl radicals).

Challenging IPEC-J2 cells with *S.* Typhimurium or *E. coli* led to a significant elevation of IL-6 and IL-8 production—a finding also revealed by numerous earlier investigations [12,53,63]. In our experiments, the use of *L. rhamnosus* for pre-treatment, co-treatment, and post-treatment effectively mitigated the *Salmonella*-triggered IL-8 secretion. Additionally, both pre-treatment and post-treatment resulted in a reduction in the increased secretion of IL-6. The probiotic strain *Lactobacillus reuteri* ATCC 53608 demonstrated a decrease in *Salmonella*-evoked IL-8 secretion, aligning with our results showing that probiotics have the potential to alleviate the proinflammatory cytokine response during pathophysiological challenges [28]. When IPEC-J2 cells were exposed to *E. coli*, the application of *L. rhamnosus* before, simultaneously with, or after the challenge did not result in a reduction in *E. coli*-induced IL-6 production. On the contrary, some researchers found that co-incubation with probiotic bacteria (*E. faecium*) reduced the elevation of the proinflammatory cytokine IL-8 induced by *E. coli* [12,14]. Our findings indicate that the impact of *L. rhamnosus* on the proinflammatory response of IPEC-J2 cells varies depending on the specific cytokine under examination and the causative agent (*E. coli* or *S.* Typhimurium) employed to induce inflammation.

In our experiments, the inhibitory effect of *L. rhamnosus* was unaffected by the time of addition; *L. rhamnosus* significantly inhibited *S.* Typhimurium and *E. coli* adhesion across all three treatment conditions (pre-treatment, co-treatment, and post-treatment). Forestier et al. also demonstrated that the inhibiting effect of probiotic bacteria on the adhesion of pathogens does not depend on the time of addition; *Lactobacillus casei rhamnosus* reduced the adhesion of three pathogens (enteropathogenic and enterotoxigenic *E. coli* and *Klebsiella pneumoniae*), irrespective of whether the probiotic strain was introduced before, during, or after incubation with the pathogen [32]. Our discovery that pre-treatment effectively inhibits pathogen adhesion suggests that the probiotic species that we tested can effectively exclude the attachment of pathogenic bacteria. Moreover, the ability of co-treatment to impede pathogen adhesion indicates that the examined probiotics can effectively compete with the pathogens. Lastly, the success of post-treatment demonstrates that the investigated probiotics are also capable of disrupting established pathogen colonization. The presence of *L. rhamnosus* could potentially impede *E. coli* and *S.* Typhimurium’s access to tissue receptors through steric hindrance, offering a possible explanation for the reduced adhesion of these pathogens in the presence of probiotic bacteria. It is worth noting that there may be additional mechanisms at play. Pathogen adhesion might be constrained by the combined influence of probiotic bacteria and mucin. For instance, HT29 cells exhibited increased mucin production when exposed to probiotics [32]. Additionally, IPEC-J2 cells secrete mucins that could interact with the presence of *L. rhamnosus*, potentially inhibiting the adhesion of *E. coli* and *S.* Typhimurium [32]. The generation of substances with bacteriostatic and bactericidal properties could potentially have an indirect impact on adhesion inhibition. For instance, biosurfactants produced by Lactobacilli have been demonstrated to exhibit inhibitory effects against various Gram-negative and Gram-positive species, including *S.* Typhimurium and *E. coli*. This inhibitory property might also contribute to the adhesion-inhibiting effects of probiotics. However, further research is necessary to elucidate the precise mechanism through which *L. rhamnosus* exerts its adhesion-inhibiting effects [64].

## 5. Conclusions

In summary, our research has demonstrated various advantageous effects of *L. rhamnosus* in an in vitro porcine model that simulated gastrointestinal infection induced by either *E. coli* or *S.* Typhimurium. These effects encompassed antioxidative, inhibitory, anti-inflammatory, and barrier-enhancing properties. This study made a meaningful contribution to the understanding of the specific benefits associated with *L. rhamnosus*, which can be valuable when selecting a probiotic for practical applications. Nevertheless, additional investigations are required to confirm these findings through in vivo experiments.

The application of probiotics offers a solution to the challenge of finding alternative therapies that can enhance gastrointestinal well-being without resorting to using antibiotics. To achieve the most favorable outcomes, applying *L. rhamnosus* in a mixture with other probiotic species (as multi-strain or multi-species combinations) appears to be favorable. However, additional investigations are required to determine whether a blend of probiotics exerts its effects through additive, antagonistic, or synergistic mechanisms. Additionally, our findings contribute to a deeper understanding of probiotic actions on intestinal porcine epithelial cells and establish a starting point for further in vivo research and applications in both human and swine contexts. In the future, our model has the potential to be further developed to create an IPEC-J2 immune cell co-culture model, which can more precisely mimic gut complexity and allows us to study the interactions between probiotics, intestinal, and immune cells.

## Figures and Tables

**Table 1:** **Decrease in the amount of bacteria (*S.* Typhimurium or *E. coli*) adhered to IPEC-J2 cells via treatment with *L. rhamnosus*. *L. rhamnosus* PRE**: pre-treatment before bacterial infection with 10^8^ CFU/mL of *L. rhamnosus*; ***L. rhamnosus* CO**: co-treatment of bacterial infection with 10^8^ CFU/mL of *L. rhamnosus*; ***L. rhamnosus* POST**: treatment after bacterial infection with 10^8^ CFU/mL of *L. rhamnosus*. Data are shown as the bacterial count reduction compared to the mean value of the control (attached bacteria without probiotic treatment) that was considered to be 100%. *n* = 4/group. Significant difference compared to control (treatment without probiotic): * *p* < 0.05, *** *p* < 0.001 [46].

Treatment	*Escherichia coli*		*Salmonella enterica* Serovar Typhimurium	
	Reduction	*p* Value	Reduction	*p* Value
*L. rhamnosus* PRE	−90.80%	*p* < 0.001 ***	−96.33%	*p* < 0.001 ***
*L. rhamnosus* CO	−34.92%	*p* < 0.05 *	−96.76%	*p* < 0.001 ***
*L. rhamnosus* POST	−74.63%	*p* < 0.001 ***	−91.02%	*p* < 0.001 ***

## Data Availability

All data that support the above-detailed findings can be obtained from the corresponding author upon request.
